# IGF2BP2 promotes colorectal cancer progression by upregulating the expression of TFRC and enhancing iron metabolism

**DOI:** 10.1186/s13062-023-00373-x

**Published:** 2023-04-23

**Authors:** Tian-yue Liu, Chen-chen Hu, Chen-ying Han, Si-yi Mao, Wen-xin Zhang, Yi-ming Xu, Yuan-jie Sun, Dong-bo Jiang, Xi-yang Zhang, Jia-xing Zhang, Jing Wang, Xu-peng Qiao, Jing-yu Pan, Shu-ya Yang, Kun Yang

**Affiliations:** 1 Department of Immunology, the Fourth Military Medical University, Xi’an, Shaanxi 710032 China; 2 School of Basic Medicine, the Fourth Military Medical University, Xi’an, Shaanxi 710032 China; 3 Military Medical Innovation Center, the Fourth Military Medical University, Xi’an, Shaanxi 710032 China; 4 College of medical technology, Shaanxi University of traditional Chinese Medicine, Xianyang, Shaanxi 712046 China; 5 Department of Rheumatology and Immunology, Tangdu Hospital of the Air Force Medical University, Xi’an, Shaanxi 710038 China

**Keywords:** Colorectal cancer, IGF2BP2, Iron metabolism, m6A-TFRC

## Abstract

**Background:**

Colorectal cancer (CRC) is one of the most common malignant tumors of the digestive system, ranking third for morbidity and mortality worldwide. At present, no effective control method is available for this cancer type. In tumor cells, especially iron metabolization, is necessary for its growth and proliferation. High levels of iron are an important feature to maintain tumor growth; however, the overall mechanism remains unclear.

**Methods:**

We used western blotting, immunohistochemistry (IHC) and real-time quantitative PCR to analyze the expression of IGF2BP2 in cell lines and tissues. Further, RNA-sequencing, RNA immunoprecipitation and methylated RNA immunoprecipitation experiments explored the specific binding of target genes. Moreover, the RNA stability assay was performed to determine the half-life of genes downstream of IGF2BP2. In addition, the Cell Counting Kit-8, colony formation assay, 5-ethynyl-2’-deoxyuridine assay and flow cytometry were used to evaluate the effects of IGF2BP2 on proliferation and iron metabolism. Lastly, the role of IGF2BP2 in promoting CRC growth was demonstrated in animal models.

**Results:**

We observed that IGF2BP2 is associated with iron homeostasis and that TFRC is a downstream target of IGF2BP2. Further, overexpression of TFRC can rescue the growth of IGF2BP2-knockdown CRC cells. Mechanistically, we determined that IGF2BP2 regulates TFRC methylation via METTL4, thereby regulating iron metabolism and promoting CRC growth. Furthermore, using animal models, we observed that IGF2BP2 promotes CRC growth.

**Conclusion:**

IGF2BP2 regulates TFRC mRNA methylation via METTL4, thereby regulating iron metabolism and promoting CRC growth. Our study highlights the key roles of IGF2BP2 in CRC carcinogenesis and the iron transport pathways.

**Supplementary Information:**

The online version contains supplementary material available at 10.1186/s13062-023-00373-x.

## Introduction

At present, colorectal cancer (CRC) is one of the most prevalent type malignancies, ranking third for morbidity and mortality worldwide [1]. Several treatment options, including surgery, radiotherapy, chemotherapy, and combination therapy, are available for CRC, with certain effects; however, all these methods have some limitations [2]. Therefore, it is crucial to identify new and effective therapeutic targets for CRC intervention.

Insulin-like growth factor 2 mRNA-binding protein 2 (IGF2BP2), a member of IGF2BPs family, can maintain the mRNA stability [3]. It has been reported that RNA binding proteins can regulate RNA metabolism in many aspects; their imbalance can lead to the occurrence of several diseases [4]. In particular, IGF2BP2 promotes the proliferation of tumor cells [5]. However, its role in iron metabolism remains unclear. We found that IGF2BP2 is closely related to iron homeostasis by RNA-seq.

Iron is an element necessary for the human body. It participates in many protein/enzymes-related processes [6, 7]. Therefore, it is important to maintain iron homeostasis in tissues and cells, and iron-related proteins are essential for regulating iron homeostasis [8]. Furthermore, regulating the function of these proteins is necessary for maintaining iron balance and homeostasis in the body because an imbalance can lead to abnormal pathological changes. Previous studies revealed that body high iron levels are positively correlated with increased cancer risk; further, high iron levels facilitate the growth of tumor cells [9, 10].

In the present study, we found that IGF2BP2 promotes CRC cell growth and iron metabolism. Tissue microarray (TMA) staining revealed that high IGF2BP2 expression was positively correlated with CRC stage. Further, RNA-seq revealed that iron-related pathways were significantly enriched in IGF2BP2 knockout cells. In vivo and vitro experiments, knockdown of IGF2BP2 decreased CRC proliferation and iron metabolism. Mechanistically, IGF2BP2 binds to Transferrin receptor (TFRC, also called TFR1 or CD71), a key enzyme involved in iron metabolism, via N^6^-methyladenosine (m6A) modification, thereby affecting iron metabolism. TFRC, a key protein in the iron transport pathway, is abundantly expressed in various tumors [11, 12]. Furthermore, we demonstrated that the ability of iron metabolism in IGF2BP2-knockout CRC cells can be rescued by overexpressing TFRC. Taken together, we elucidated that IGF2BP2 can promote tumor proliferation and iron metabolism. Our study illustrates the function of IGF2BP2 in iron transport pathways and CRC oncogenesis and suggests a novel clinical treatment strategy for patients with CRC.

## Results

### High IGF2BP2 expression is consistent with CRC tumor stage

**Fig. 1:**
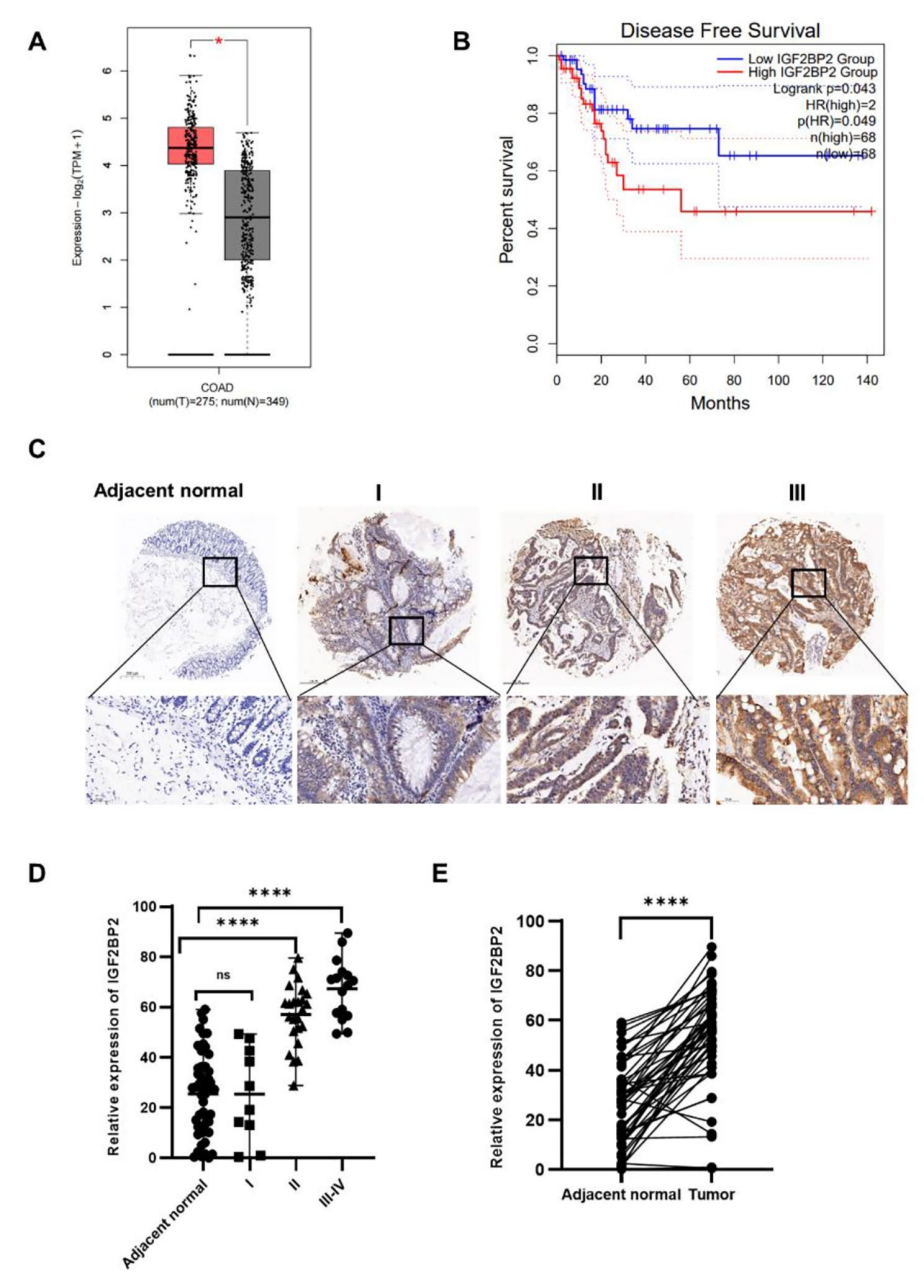
IGF2BP2 is highly expressed in colorectal cancer and correlates with the tumor stage. (**A, B**) Differences in IGF2BP2 expression between COAD tissue and normal tissue were analyzed using the GEPIA database (http://gepia.cancer-pku.cn/detail.php). (**C**) IGF2BP2 expression in para-carcinoma tissues and COAD tumor tissues at various clinical stages as determined by IHC analysis. (**D**) IGF2BP2 expression in 50 COAD tumor tissue samples (10 samples were stage I, 24 were stage II, and 16 were stage III-IV). (Student’s t- test). (**E**) IGF2BP2 expression in colorectal cancer tissues and adjacent normal tissues (n = 50). (paired t- test). *P < 0.05 and **** *P* < 0.0001

To determine the role of IGF2BP2 in CRC, we analyzed the data of CRC and normal tissues in the Gene Expression Profiling Interactive Analysis (GEPIA) database (http://gepia.cancer-pku.cn/detail.php), and observed that IGF2BP2 was highly expressed in CRC and positively correlated with poor prognosis (Fig. 1A, B). This result is consistent with previous reports [13]. Further, we performed immunohistochemical (IHC) staining of IGF2BP2 in 50 CRC patient tissues, using matched adjacent normal tissues as the matched group, to elucidate its expression pattern. IGF2BP2 expression was higher in CRC tissues than in adjacent normal tissues, and this expression was positively correlated with clinical stage (Fig. 1C, D). Paired analysis of these 50 cancer and adjacent tissues revealed that IGF2BP2 is highly expressed in CRC (Fig. 1E). Moreover, we determined the correlation between the clinical characteristics of patients with CRC and IGF2BP2 expression. Then, we divided the patients into high and low-expression groups based on their median IGF2BP2 expression. Statistical analysis revealed that IGF2BP2 expression was consistent with the degree of malignancy (i.e., tumor size, lymph node metastasis, and stage; Table 1).

**Table 1:** IGF2BP2 expression and clinicopathological characteristics of patients with colorectal cancer

Characteristics	IGF2BP2 low experssion		IGF2BP2 high experssion			*P*	
	N	%	N	%			
Gender							
Male	9	18.0	13	26.0	0.2545		
Female	16	32.0	12	24.0			
Age(years)							
< 56	11	22.0	13	26.0	0.5661		
> 56	14	28.0	12	24.0			
Tumor size							
T1 or T2	10	20.0	2	4.0	0.0081*		
T3 or T4	15	30.0	23	46.0			
Lymph node							
N0	22	44.0	13	26.0	0.0176*		
N1	3	6.0	10	20.0			
N2	0	0	2	4.0			
Grade							
1	3	6.0	3	6.0	0.2822		
2	11	22.0	19	38.0			
2–3	4	8.0	1	2.0			
3	3	6.0	2	4.0			
Stage							
I	10	20.0	0	0	0.0008*		
II	11	22.0	13	26.0			
III-IV	4	8.0	12	24.0			

* indicates *P* < 0.05

### Downregulation of IGF2BP2 inhibits the proliferation of CRC cell lines

**Fig. 2:**
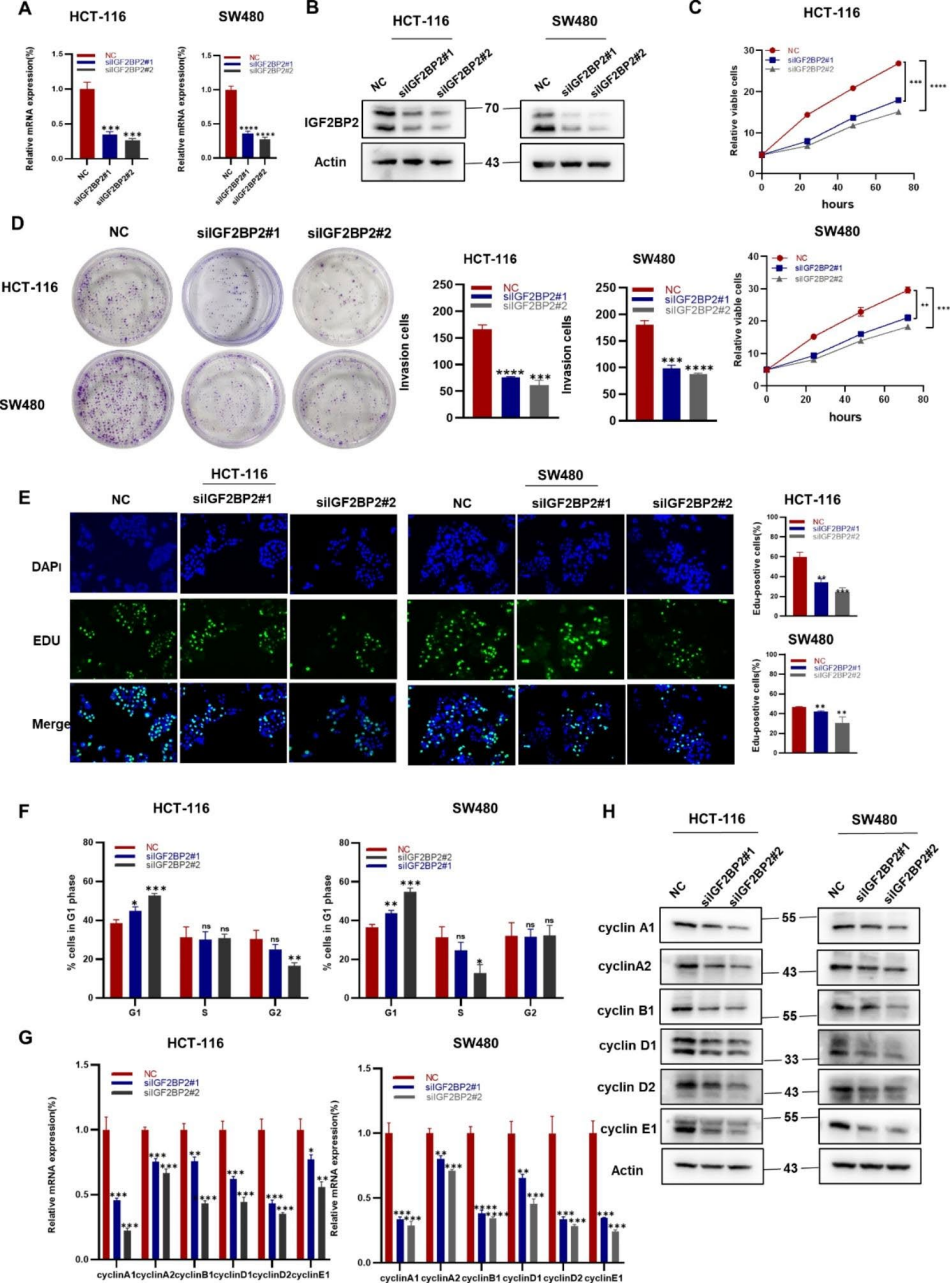
Downregulation of IGF2BP2 inhibits the proliferation and cell cycling of colorectal cancer cell lines. (**A, B**) HCT-116 and SW480 cells were transfected with IGF2BP2 siRNA, and then IGF2BP2 mRNA (**A**) and protein (**B**) levels were determined by qPCR and western blotting, respectively. (**C-E**) After IGF2BP2 silencing, the viability (**C**), colony forming ability (**D**), and proliferative ability (EdU assay) (**E**) of the cells were evaluated. Scale bars: 150 μm. (**F, G, H**) The effect of IGF2BP2 on the cell cycle. (**F**), HCT-116 and SW480 cells were transfected with the IGF2BP2 siRNA and subjected to flow cytometry after propidium iodide (PI) staining. After transfection, the cell cycle-related gene expression and protein levels were assessed using qRT-PCR (**G**) and western blotting (**H**), respectively. Each value represents the mean ± SD for triplicate samples (Student’s t-test). *P < 0.05, **P < 0.01, ***P < 0.001 and **** *P* < 0.0001

To explore the effect of IGF2BP2 on CRC, an siRNA was used to knock down IGF2BP2 in HCT-116 and SW480 cells. The transfection efficiency of the IGF2BP2 siRNA was measured using qRT-PCR (Fig. 2A) and western blotting (Fig. 2B). The CCK8, colony formation and EdU assay revealed that cell proliferation was weakened after IGF2BP2 Knockdown (Fig. 2C, D and E). Furthermore, depletion of IGF2BP2 causes cell arrest in the G1 phase (Fig. 2F). In addition, cell cycle-related genes and proteins decreased after the IGF2BP2 knockdown (Fig. 2G, H). Taken together, these results suggest that IGF2BP2 can promote CRC cell proliferation by inducing cell cycle progression.

### IGF2BP2 promotes iron metabolism in CRC cells

**Fig. 3:**
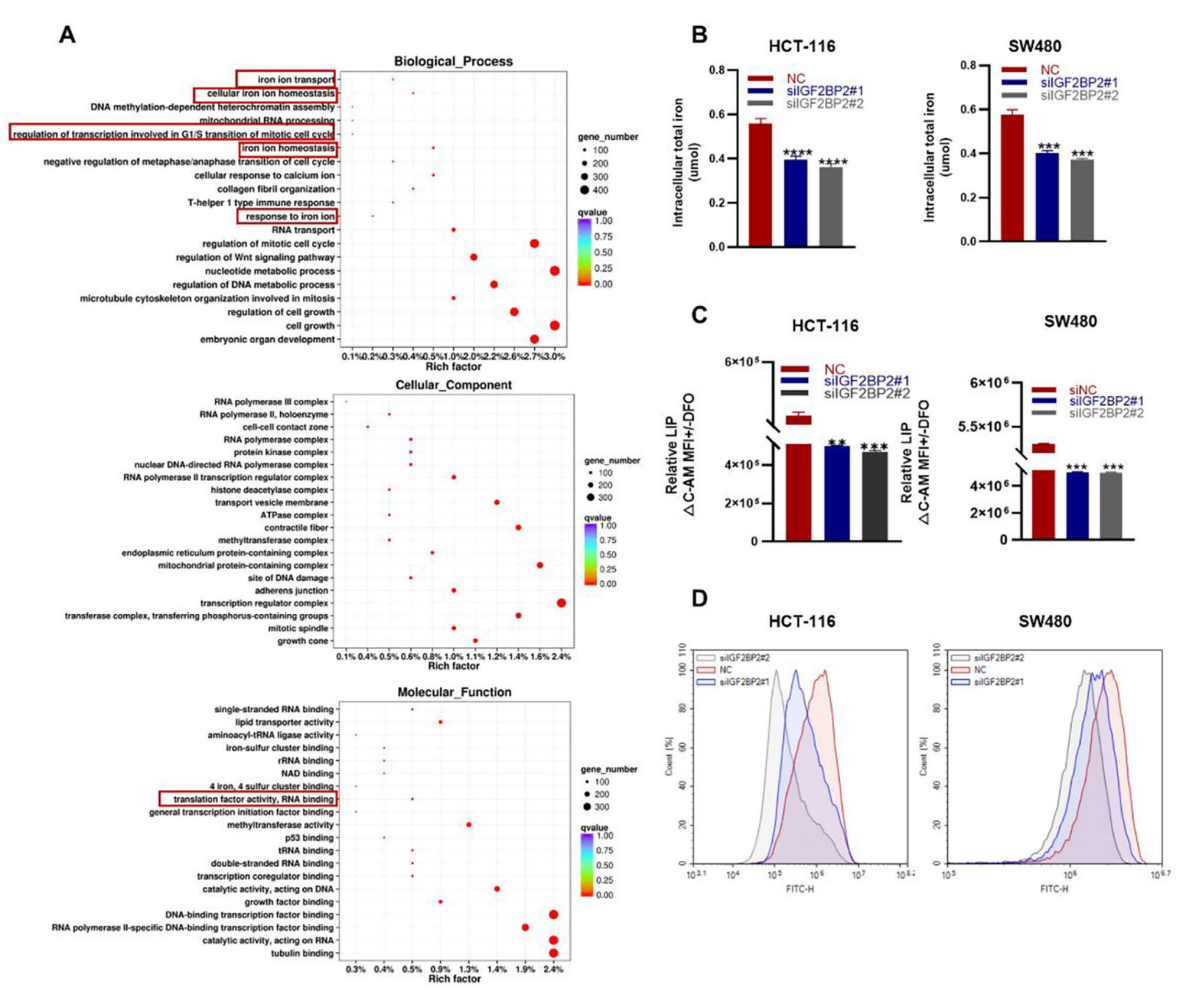
IGF2BP2 promotes iron metabolism in colorectal cancer. (**A**) Gene Ontology (GO) analysis of the 20 relevant pathways in HCT-116 cells enriched using RNA-seq. (**B**) Intracellular iron levels were measured using an iron assay kit after transfecting HCT-116 and SW480 cell lines with IGF2BP2 siRNA. (**C**) Calcein-AM (C-AM) was used to quantify cellular LIP levels via spectrophotometric measurements at an absorbance wavelength of 525 nm. Subtract the mean fluorescence intensity (MFI) of C-AM from the MFI of C-AM treated with DFO. (**D**) ROS content in HCT-116 and SW480 cells were measured after IGF2BP2 knockdown. Each value represents the mean ± SD for triplicate samples (Student’s t-test). **P < 0.01, ***P < 0.001 and **** *P* < 0.0001

To elucidate the function of IGF2BP2 in CRC cells, we evaluated the data from The Cancer Genome Atlas (TCGA). Interestingly, IGF2BP2 was associated with RNA binding, iron homeostasis, and cell cycle pathways (Figure S1). Next, we performed transcriptome sequencing (RNA-Seq) of HCT-116 cells after IGF2BP2 knockdown. The results revealed that RNA binding, iron homeostasis, cell cycle pathways were significantly altered compared with the NC group; however, its ranking was not the first. A Possible reason for this result is that a relatively small number of genes are involved in the iron metabolism pathway (Fig. 3A). As previous study reported that both the IGF2BP2 mRNA-binding protein family and YTHDF family are m6A readers [14]. In head and neck squamous cell carcinoma, YTHDF1 regulates iron metabolism by methylating TFRC (m6A methylation) [14]. The function of IGF2BPs may be similar to that of the YTHDF family. To confirm this hypothesis, we evaluated the changes in iron metabolism-related indicators. IGF2BP2 knockdown significantly decreased total iron levels in CRC cells (Fig. 3B). Labile iron pool (LIP) is derived from extracellular transport or ferritin release. It accumulates in cells and produces reactive oxygen species (ROS) [9, 10]. IGF2BP2 knockdown significantly decreased intracellular LIP levels, indicating a decrease in Fe^2+^ content. (Figs. 3 C); Simultaneously, ROS was reduced (Fig. 3D). Taken together, these results suggest that IGF2BP2 affects iron metabolism in CRC cells.

### IGF2BP2 promotes CRC growth by regulating iron metabolism-related genes

**Fig. 4:**
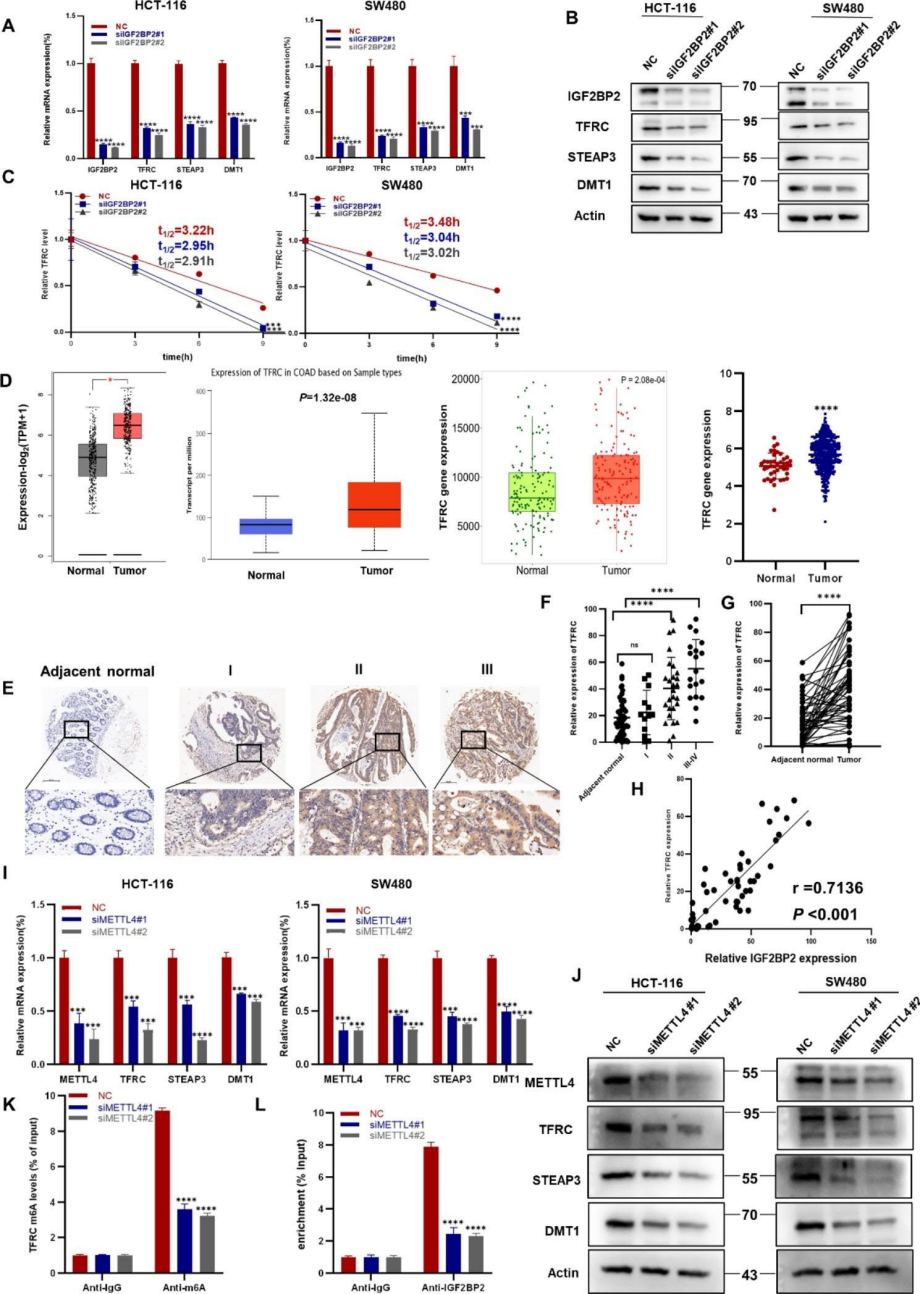
IGF2BP2 participates in colorectal cancer growth by regulating the expression of iron metabolism-related genes. (**A, B**) qRT-PCR (**A**) and western blotting (**B**) of the potential target genes and their products in HCT-116 and SW480 cells after IGF2BP2 knockdown. (**C**) The decay rate of mRNA and qPCR of TFRC at the indicated time points after treating HCT-116 and SW480 cells with actinomycin D (5 µg/ml) after IGF2BP2 inhibition. (**D**) Differences in TFRC expression between COAD tissue and normal tissue were analyzed using the GEPIA ((http://gepia.cancer-pku.cn/detail.php)), UALCAN (http://ualcan.path.uab.edu/), TNMplot (https://tnmplot.com/analysis/) and UCSC (https://xena.ucsc.edu/) databases. (**E**) The expression of TFRC in para-carcinoma tissues and CRC tumor tissues at various clinical stages as determined via IHC analysis. (**F**) Expression of TFRC in different stages of colorectal cancer and adjacent normal tissues (n = 50). (**G**) TFRC expression in colorectal cancer tissues and adjacent normal tissues (n = 50). (**H**) Correlation between IGF2BP2 and TFRC expression in COAD in tissue microarrays. (**I, J**) Relative mRNA expression of METTL4, an iron metabolism gene, was determined using qRT-PCR (**I**) and western blotting (**J**). (**K**) m6A-RIP assay was performed to detect m6A levels in TFRC mRNA in HCT-116 cells after METTL4 silencing. (**L**) Binding of IGF2BP2 to TFRC was verified using the RIP assay. Each value represents the mean ± SD for triplicate samples (Student’s t-test). *P < 0.05, ***P < 0.001 and **** *P* < 0.0001

Previous studies have reported that after binding to TF, TFRC is needed for entry into peripheral tissues. This complex is then endocytosed into endosomes, where Fe^3+^ is released. Six transmembrane epithelial antigen proteins (STEAP) of the prostate help convert Fe^3+^ to Fe^2+^, which is exported to the cytoplasm via divalent metal transporter 1 (DMT1), resulting in Fe^2+^ accumulation in LIP [7, 15]. To explore the molecular mechanism by which IGF2BP2 is involved in CRC progression, we knocked down IGF2BP2 in HCT-116 and SW480 cells and determined the changes in the expression of iron metabolism-related genes. The mRNA expression and protein levels of iron metabolism-related genes, such as TFRC, STEAP3 and DMT1 were decreased in CRC cells (Fig. 4A, B). As an RNA binding protein, IGF2BP2 can regulate transcription at the mRNA level. We selected HCT-116 cells for the RNA immunoprecipitation (RIP) assay and found that IGF2BP2 can bind to TFRC (Figure S2). In addition, mRNA stability was determined in IGF2BP2 knockdown and control HCT-116 and SW480 cells. As hypothesized, IGF2BP2 knockdown reduced the half-life of TFRC mRNA (Fig. 4C). A study has reported that TFRC expression correlates with cellular iron load [16]. TFRC expression was further analyzed using the GEPIA, University of ALabama at Birmingham CANcer data analysis Portal (UALCAN), TNMplot, and UCSC Xena databases. CRC tissues were higher than normal tissues in TFRC expression (Fig. 4D). IHC staining of CRC tissue microarray showed the same results and TFRC expression was positively correlated with CRC stage (Fig. 4E, F). Then we compared CRC tissues to adjacent normal tissues, the former was stronger (Fig. 4G; Table 2). The scatter plot demonstrated that IGF2BP2 is positively correlated with TFRC (Fig. 4H). METTL4 is a type of m6A-modified RNA that regulates tumor progression by modifying RNA [17]. After METTL4 knockdown in CRC cells, the expression of iron metabolism-related genes was significantly reduced, particularly TFRC (Fig. 4I, J). Furthermore, we performed the m6A-RIP assay using METTL4 knockdown HCT-116 cells. qRT-PCR revealed that m6A-modified TFRC was significantly down-regulated after METTL4 knockdown (Fig. 4K). RIP revealed that IGF2BP2-bound TFRC mRNA expression was decreased in METTL4- silenced HCT-116 cells (Fig. 4L). Taken together, the results suggest that IGF2BP2 regulates TFRC mRNA methylation via METTL4, thereby regulating iron metabolism.

**Table 2:** TFRC expression and clinicopathological characteristics of patients with CRC

Characteristics	TFRC low experssion		TFRC high experssion		*P*
	N	%	N	%	
Gender					
Male	11	22.0	11	22.0	> 0.9999
Female	14	28.0	14	28.0	
Age(years)					
< 56	13	26.0	12	24.0	0.7773
> 56	12	24.0	13	26.0	
Tumor size					
T1 or T2	9	45.0	3	6.0	0.0469*
T3 or T4	16	32.0	22	44.0	
Lymph node					
N0	21	42.0	13	26.0	0.0502
N1	3	6.0	10	20.0	
N2	1	2.0	2	4.0	
Grade					
1	3	6.0	3	6.0	0.3080
2	17	34.0	17	34.0	
2–3	4	8.0	1	2.0	
3	1	2.0	4	8.0	
Stage					
I	9	18.0	1	2.0	0.0055*
II	12	24.0	12	24.0	
III-IV	4	8.0	12	24.0	

* indicates *P* < 0.05

### TFRC overexpression partially rescues IGF2BP2 knockdown-induced CRC cells proliferation and iron metabolism

**Fig. 5:**
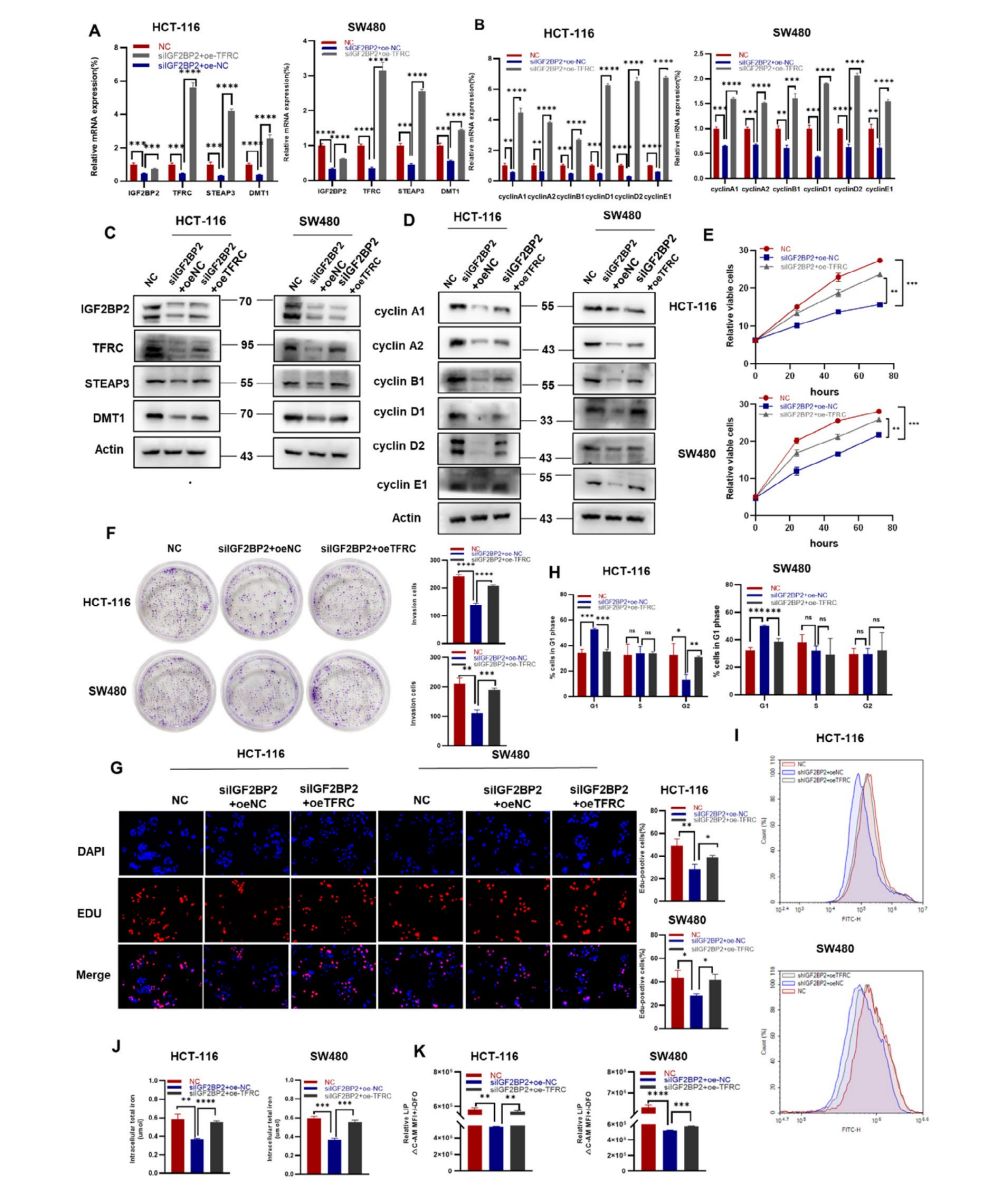
TFRC overexpression partially rescues IGF2BP2 knockdown-mediated attenuation of colorectal cancer cell proliferation and iron metabolism. (**A, B**) After transfecting TFRC expression plasmids, the mRNA expression of TFRC and downstream factors and cell cycle-related factors were assessed using qRT-PCR. (**C, D**) Transfection of HCT-116 and SW480 cells expressing shIGF2BP2 with TFRC, and determination of protein levels using western blot. (**E, F, G**) Cell viability, clonogenicity, and proliferative capacity of shIGF2BP2-expressing HCT-116 and SW480 cells transfected or not transfected with oe-TFRC as assessed using the CCK-8 assay (**E**), colony formation assay (**F**), and EdU staining (**G**), respectively. (**H**) Assessment of plasmid-transfected HCT-116 and SW480 cells for TFRC via propidium iodide (PI) staining using flow cytometry. (**I, J, K**) Overexpression of TFRC in HCT-116 and SW480 cells transfected with shIGF2BP2 and determination of reactive oxygen species (ROS) concentration (**I**), total iron content (**J**), and labile iron pool (LIP) content (**K**). Each value represents the mean ± SD for triplicate samples (Student’s t-test). *P < 0.05, **P < 0.01, ***P < 0.001 and **** *P* < 0.0001

Because TFRC may be a targeting downstream of IGF2BP2, we transfected IGF2BP2 knockdown HCT-116 and SW480 cells with TFRC-overexpressing plasmids. We found that the mRNA expression and protein levels of iron metabolism and cell cycle-related genes were restored (Fig. 5A and B, 5C and D). Further, TFRC overexpression partially restored the viability and colony-forming ability of IGF2BP2 knockdown cells (Fig. 5E, F). Moreover, the EdU assay revealed that TFRC overexpression rescued the proliferative ability in IGF2BP2-knockdown cells (Fig. 5G). Simultaneously, flow cytometry of the cell cycle obtained similar results (Fig. 5H). To explore the effect of TFRC on iron metabolism in CRC cells, we detected iron metabolism indicators such as total iron, LIP, and ROS. TFRC overexpression partially restored, the intracellular total iron content, LIP levels and ROS content compared with the IGF2BP2 knockdown group (Fig. 5J, K and 5I). Taken together, these results suggest that the proliferation and iron metabolism of CRC cells, attenuated by IGF2BP2 silencing, are partially restored by the TFRC expression. Therefore, TFRC is a key molecule in CRC development and functions as a downstream target of IGF2BP2.

### IGF2BP2 enhances CRC growth in vivo

**Fig. 6:**
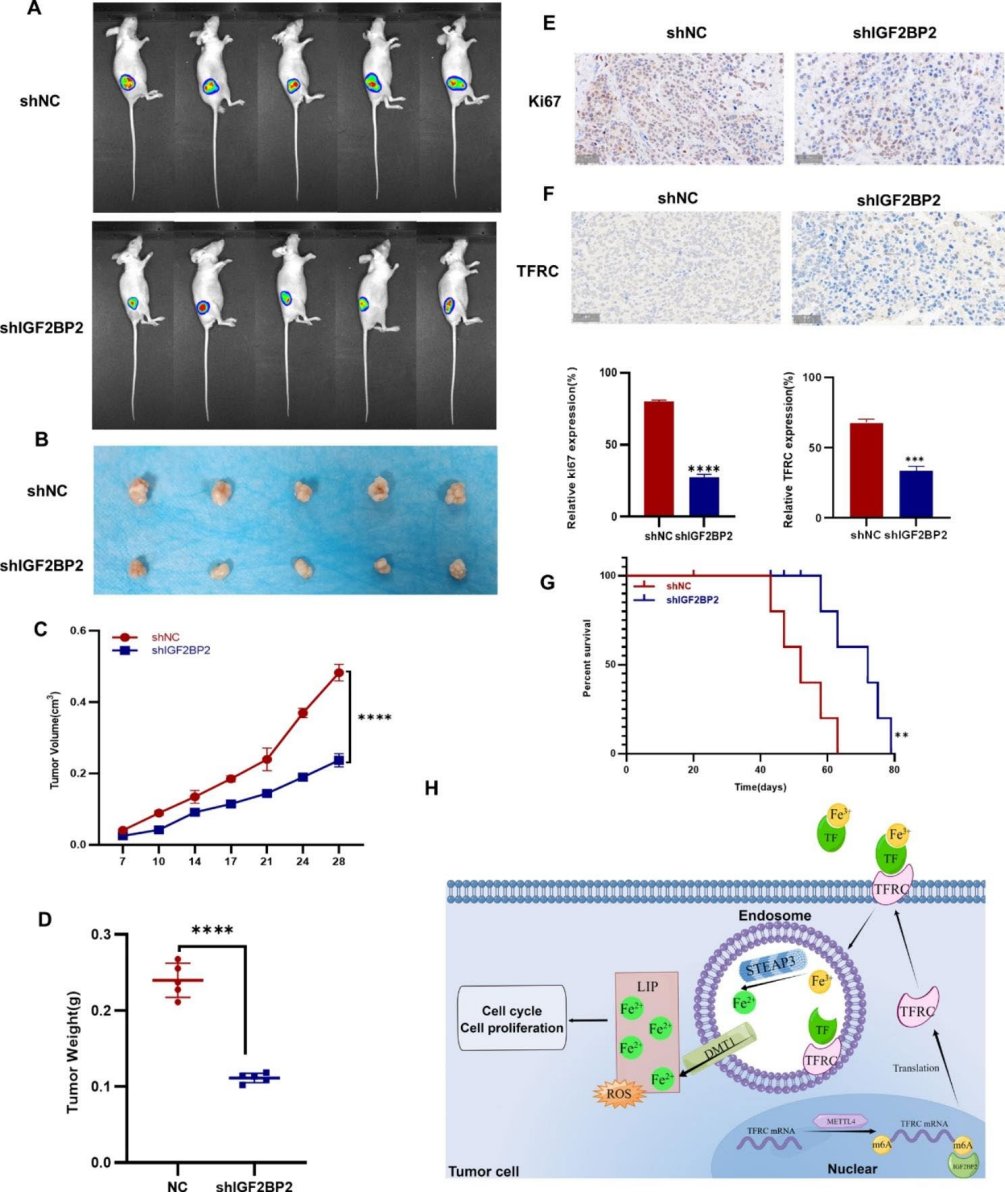
IGF2BP2 enhances colorectal cancer tumor growth in vivo. (**A, B**) HCT-116 cells stably expressing shNC, shIGF2BP2 were subcutaneously injected into the right side of nude mice. Tumor images were obtained using the PerkinElmer IVIS preclinical in vivo imaging system at 25 (**A**) and 28 (**B**) days after injection. (**C**) Tumor volumes were measured after every 3–4 days starting on the 7th day after injection. (**D**) The weight of the xenografted tumors was measured. (**E**) Tumor sections were subjected to IHC staining with an antibody against Ki-67. Scale bar: 50 μm. (**F**) Tumor sections were subjected to IHC staining with an antibody against TFRC. Scale bar: 50 μm. (**G**) Kaplan-Meier survival curve shows the overall survival rate of mice in each group (n = 5, *p < 0.05, using the log-rank test). (**H**) A proposed regulatory model depicting the role of IGF2BP2 in iron metabolism and tumorigenesis (By Figdraw). Each value represents the mean ± SD for triplicate samples (Student’s t-test). **P < 0.01 and **** *P* < 0.0001

A mouse model was constructed to determine the role of IGF2BP2 in vivo. NC and IGF2BP2 knockdown HCT-116 cells were subcutaneously injected into 6-week-old nude mice. Changes in tumor size in mice were observed and recorded after every 3–4 days, and the tumor was imaged in vivo on the 25th day. Mice injected with shIGF2BP2-expressing HCT-116 cells had significantly smaller tumors than those injected with shNC cells (Fig. 6A). On the 28th day, mice were euthanized and tumors were excised. Compared with the NC group, the tumor in the shIGF2BP2 group was smaller and lighter (Fig. 6B, C and 6D). Subsequently, Ki-67 immunohistochemical staining was performed on tumor tissues to evaluate tumor proliferation; the proliferation of the shIGF2BP2 group was attenuated (Fig. 6E). Furthermore, after down-regulating IGF2BP2, IHC staining of mouse tumor tissue sections with the TFRC antibody revealed that TFRC expression decreased after IGF2BP2 was down-regulated (Fig. 6F). Next, we analyzed prepared a survival curve and observed that the survival time of the shIGF2BP2 group was increased (Fig. 6G). Figure 6H presents the proposed regulatory model of IGF2BP2 in CRC, in which IGF2BP2 promotes iron metabolism and tumor progression in CRC by regulating METTL4, thereby regulating the methylation of TFRC mRNA.

## Discussion

CRC has been named as the third leading cause of cancer deaths, with an increasing annual incidence rate [1, 18]. At present, there are limited treatment methods for CRC, therefore more effective treatment strategies are warranted for intervention. For the first time, the present study investigated the potential function of IGF2BP2 in CRC progression and explored the role of IGF2BP2 in promoting iron metabolism in CRC. The results revealed that targeting metabolic pathways may be a potential strategy for CRC treatment.

IGF2BPs are a family of carcinoembryonic proteins that regulate cellular function in cancers and act as m6A readers. Further, they are involved in replication and transcription in various cancers [19, 20]. IGF2BPs can maintain mRNA stability via m6A modifications [21–23]. Knockdown of the lncRNA LINRIS attenuates IGF2BP2-regulated aerobic glycolysis in CRC [23]. IGF2BP2 promotes the liver cancer growth via FEN1[24]; however, its role in iron metabolism remains unclear. In the present study, we analyzed TCGA database and observed that IGF2BP2 and TFRC expression was generally increased in CRC tissues; this result, was consistent with our clinical sample data. Furthermore, we elucidated the relationship between iron metabolism and IGF2BP2. Using Gene Ontology enrichment analysis and clinical samples, we concluded that IGF2BP2 is positively correlated with TFRC. The RIP and m6A-RIP assays revealed that IGF2BP2 can bind to TFRC to regulate iron metabolism. After IGF2BP2 knockdown in CRC cells, the expression of the iron metabolism-related genes TFRC, STEAP3, and DMT1 as well as the total iron content and ROS level were significantly decreased. Simultaneously, TFRC overexpression rescued the decrease in iron levels and inhibition of CRC progression caused by IGF2BP2 knockdown. Our results demonstrate for the first time that IGF2BP2 regulates iron metabolism by regulating TFRC.

Iron is crucial for cell growth and survival. Its dysregulation can disrupt iron homeostasis. A large amount of iron is required to maintain tumor cell growth; therefore, iron balance is important for cell growth and survival [25, 26]. TFRC is essential for balancing iron homeostasis in the body [27]. We found that IGF2BP2 enhances TFRC protein levels in CRC cells, thereby increasing iron uptake. Ultimately, iron is used for cell proliferation and to meet metabolic demands; therefore, it plays a major role in metabolism. How IGF2BP2 regulates TFRC may be a watershed event in iron metabolism and tumor progression. The interaction between two different oncogenes is essential for both cancer development and new therapeutic strategies.

In conclusion, in the present study, we elucidated that IGF2BP2 expression is upregulated in CRC cells, which is consistent with clinical prognosis. Further, IGF2BP2 is closely related to iron metabolism in CRC. In addition, IGF2BP2 can bind to TFRC to regulate iron metabolism and enhance tumor growth and proliferation. From the perspective of treatment and prevention, targeting IGF2BP2 and TFRC may become novel strategies for CRC diagnosis and treatment.

## Materials and methods

### Cell culture

The human CRC cell line, HCT-116 was cultured in McCoy’s 5 A (Procell, China) supplemented with 10% FBS (Gibco, USA). SW480 were cultured in L15 (Meilunbio, China) supplemented with 10% FBS and incubated without CO_2_. Both cell lines were confirmed to be mycoplasma-negative.

### Transfection and lentiviral infection of cell lines

Lipofectamine 3000 (Invitrogen, USA), was used for IGF2BP2-siRNA (GenePharma, Shanghai, China) transfection. After 48 or 72 h of transfection, cells were analyzed by qRT-PCR or western blotting to confirm the transfection efficiency. Then, the IGF2BP2-shRNA lentivirus was inserted into the pds401_pL-U6-shRNA-Luc- ccdB-puro vector (TSINKGE, Beijing, China), and the infected CRC cells were grown in a culture medium containing 5 µg/ml polybrene. IGF2BP2-knockdown CRC cells were rescued and virus-infected cells were transfected using a TFRC- overexpressing plasmid. The stably transfected cell lines were selected using 10 µg/ml puromycin. All the sequences mentioned above are presented in Table S1.

### Quantitative real-time PCR (qRT-PCR) and RNA-seq

Total RNA was extracted from the cells using an RNA isolation kit (TSINKGE, Beijing, China) and cDNA was obtained using the OneStep RT-PCR kit. qRT-PCR was performed using the SYBR Green PCR Master Mix (Vazyme Biotech, Nanjing, China). Table S2 lists the primer sequences.

IGF2BP2 siRNA-transfected HCT-116 cells were collected and sent to Genergy BO-Technology for sequencing.

### Western blotting

Western blotting was performed to determine the protein levels as per the method detailed described in a previous study [28]. Cells were lysed with RIPA buffer (Beyotime, China). Then, the supernatant was collected after centrifugation (4℃, 10 min) and denatured at 95 °C for 5 min before cooling on ice. Following quantification using the BCA quantification kit (Thermo Fisher Scientific, USA). Samples were separated according to different molecular weights using 10% SDS-PAGE. A PVDF membrane (Millipore, USA) was immersed in 5% skimmed milk as a blocking buffer for 1 h after transferred. Then it incubated with primary antibodies at 4℃ overnight, therewith secondary antibodies. The PVDF membrane was then developed and photographed. Antibodies are shown in the table S3 below.

### RNA stability assay

Transfected CRC cell lines were cultured in 12-well plates. After adherence, cells were treated with 5 µg/mL actinomycin for 0, 3, and 6 h [29]. Then, total RNA was isolated using a kit (TSINKGE, Beijing, China). qRT-PCR was performed to determine, the mRNA expression and half-life of each group at the indicated times points.

### EdU incorporation (5-Ethynyl-2′-deoxyuridine) assay

Cells were incubated for 2 h with 10 µM EdU reagent (Beyotime, China). Then, the treated cells were fixed with 4% PFA for 15 min and then stained according to the instructions mentioned in the kit. Photographs were taken using fluorescence microscope (EVOS M5000, Thermo Fisher Scientific, USA).

### Cell viability and colony formation assays

To measure cell viability, 8000 treated cells were seeded into per well of 96-well plates for Cell Counting Kit-8 (CCK-8) assay (Yisheng, China) to monitor cell viability. The absorbance was measured at 450 nm after incubation for 90 min with the CCK-8 reagent.

For the colony formation assay, 400 treated cells were seeded into per 60-mm dish. After 2 weeks cultured, cells were fixed with methanol for 10 min. On the top of crystal violet stained (Solarbio, Beijing, China), the cells were counted ImageJ software.

### RNA immunoprecipitation (RIP) assay

As per a previously described method [30], HCT- 116 cells were seeded into 15-cm dishes and harvested after reaching 90% confluency. Then, cells were lysed using the RIP-Assay Kit (MBL Life Science, Japan). The supernatant was added into a nuclease-free EP tube containing protein A/G PLUS-Sepharose beads and incubated at 4℃. After 1 h, the EP tube was rotated and the new supernatant was added into another EP tube containing protein A/G PLUS-Sepharose beads conjugated with antibodies (IGF2BP2 or IgG). Then, the tubes were incubated for 3 h at 4℃. Finally, antibody-immobilized bead-RNP complexes were harvested and RNA was purified and analyzed using qRT-PCR.

### m6A-RNA immunoprecipitation (m6A-RIP) assay

m6A-modified TFRC was pulled down with N6-methyladenosine (m6A) antibody (Active Motif, China) for m6A-RIP. We used mRNA Isolation Kit (NEB, China) to extract and purify total RNA of HCT-116 cells. Then we prepared the IP buffers as literature described [31]. Protein A/G PLUS-Sepharose beads were used to antibody-immobilized (m6A antibody or IgG) in the IP buffer. The purified RNA was then transferred into EP tubes containing RNase and protease inhibitors and incubated overnight at 4℃. Finally, qRT-PCR analysis was performed after RNA extraction. Table S2 lists the primers used in this assay.

### Determination of iron content

Intracellular iron (Fe^3+^ and Fe^2+^) levels were determined using the Iron Assay Kit (BIOVISION, USA). After transfecting the cells for 48 h in a 0.6-cm dish, the adherent HCT-116 or SW480 cells were lysed in buffer. Then, the lysate was sonicated, and centrifuged at 16,000×g for 10 min. Each reagent was then added as per the sequence mentioned in the instruction, and then measured the absorbance at 563 nm.

### Measurement of labile iron protein assay

LIP was measured as per the method described in a previous study [32]. Briefly, the treated cells were harvested and 0.05 µM calcein acetoxymethyl ester (Beyotime, China) was added. After incubation for 15 min at 37℃, the cells were washed twice with PBS. Then, 100 µM DFO (Absin, China) was added to the cells, and cells were incubated at 37℃ for 1 h. The control group was not treated with DFO. Flow cytometry was performed at an excitation wavelength of 488 nm and an emission wavelength of 525 nm. The difference between the groups treated with and without DFO was estimated as the LIP content.

### Flow cytometry

When treated properly, cells were collected. Then ice-cold PBS was used to wash cells and fixative containing 70% ethanol was used to fix cells at 4℃ overnight. All steps were performed as per instructions of the PI/RNase staining buffer (BD, USA). Data collection and analysis were performed using NovoExpress software (ACEA Biosciences, USA).

### Determination of ROS

Intracellular ROS was determined using the reactive oxygen detection kit (Beyotime, China). Briefly, cells were collected after transfection, and 2’-7’-dichlorofluorescein diacetate (DCFH-DA, 10 µM) was added to a serum-free medium and incubated at 37℃ for 20 min. After washing the cells three times with phosphate buffered saline (PBS), ROS content was determined using a flow cytometer (ACEA Biosciences, USA).

### Immunohistochemistry (IHC)

CRC tissue microarray (TMA) staining with antibodies targeting TFRC (1:100, Immunoway), IGF2BP2 (1:50, Proteintech) and Ki-67 (1:300, Abcam) was performed using a previously described method [33]. Stained sections were scanned and analyzed using the 3DHISTECH imaging system (Hungary). Scanning was performed using Indica Labs software (USA), results analyzed using the Aipathwell image analysis system, and calculate the H-score value according to H-score = Σ (pi × i) = (percentage of weak intensity × 1) + (percentage of moderate intensity × 2) + (percentage of strong intensity × 3).

### Animal experiments

Our animal experiments were admitted by The Ethics Committee of the Fourth Military Medical University. We chose female BALB/c nude mice for experiments. 40 mice were used in experiments. 2 × 10^5^ shNC (HCT-116 cells) cells and shIGF2BP2 (IGF2BP2 knockdown HCT-116 cells) cells were injected into right back of each nude mouse. After implantation, tumors were measured twice a week as V = 1/2×length×width^2^. Tumor images were measured using PerkinElmer IVIS.

### Statistical analysis

Experiments were repeated a least three times assurance. When there was comparation about two groups, Student’s t-test was performed using GraphPad Prism 8.0.2. For multiple groups, one-way analysis of variance was used. The Kaplan-Meier was used to describe the survival curve. The data were presented as mean ± standard deviation. *P < 0.05, **P < 0.01, ***P < 0.001, and ****P < 0.0001 were considered statistically significant. NS indicated not significant.

## Electronic supplementary material

Below is the link to the electronic supplementary material.

Supplementary Material 1

## Data Availability

All data generated or analyzed during this study are included in this published article [and its supplementary information files].

## Abbreviations

IGF2BP2: Insulin-like growth factor 2 mRNA-binding protein 2
CRC: Colorectal cancer
TFRC (TFR1): Transferrin receptor protein 1
STEAP3: Six-transmembrane epithelial antigen of the prostate 3
DMT1: Divalent metal transporter-1
LIP: Labile iron pool
DFO: Deferoxamine
ROS: Reactive oxygen species
