# Supplementary material for: IGF2BP2 promotes colorectal cancer progression by upregulating the expression of TFRC and enhancing iron metabolism
Source: Biol Direct. 2023 Apr 23;18:19. doi: 10.1186/s13062-023-00373-x (PMC10122817; doi:10.1186/s13062-023-00373-x)
Supplement: Supplementary file 1 — Supplementary Material 1 [file 13062_2023_373_MOESM1_ESM.docx]

***Supplementary Table 1. The sequences of siRNAs, shRNAs and plasmids***

| Plasmids | sequences (5' > 3') |
| --- | --- |
| siIGF2BP2#1  si/shIGF2BP2#2  siMETTL4#1  SiMETTL4#2  NC  pds401_pL-U6-shRNA-Luc-ccdB-puro/TFRC | UGAAGCUGGAAGCGCAUAUTT  CCCGCAUCAUCACUCUUAUTT  GGAGUUCACUACUUCUGUUTT  GGAUGCUAUGGAAUACCAUTT  UUCUCCGAACGUGUCACGUTT  gctagcATGATGGATCAAGCTAGATCAGCATTCTCTAACTTGTTTGGTGGAGAACCATTGTCATATACCCGGTTCAGCCTGGCTCGGCAAGTAGATGGCGATAACAGTCATGTGGAGATGAAACTTGCTGTAGATGAAGAAGAAAATGCTGACAATAACACAAAGGCCAATGTCACAAAACCAAAAAGGTGTAGTGGAAGTATCTGCTATGGGACTATTGCTGTGATCGTCTTTTTCTTGATTGGATTTATGATTGGCTACTTGGGCTATTGTAAAGGGGTAGAACCAAAAACTGAGTGTGAGAGACTGGCAGGAACCGAGTCTCCAGTGAGGGAGGAGCCAGGAGAGGACTTCCCTGCAGCACGTCGCTTATATTGGGATGACCTGAAGAGAAAGTTGTCGGAGAAACTGGACAGCACAGACTTCACCGGCACCATCAAGCTGCTGAATGAAAATTCATATGTCCCTCGTGAGGCTGGATCTCAAAAAGATGAAAATCTTGCGTTGTATGTTGAAAATCAATTTCGTGAATTTAAACTCAGCAAAGTCTGGCGTGATCAACATTTTGTTAAGATTCAGGTCAAAGACAGCGCTCAAAACTCGGTGATCATAGTTGATAAGAACGGTAGACTTGTTTACCTGGTGGAGAATCCTGGGGGTTATGTGGCGTATAGTAAGGCTGCAACAGTTACTGGTAAACTGGTCCATGCTAATTTTGGTACTAAAAAAGATTTTGAGGATTTATACACTCCTGTGAATGGATCTATAGTGATTGTCAGAGCAGGGAAAATCACCTTTGCAGAAAAGGTTGCAAATGCTGAAAGCTTAAATGCAATTGGTGTGTTGATATACATGGACCAGACTAAATTTCCCATTGTTAACGCAGAACTTTCATTCTTTGGACATGCTCATCTGGGGACAGGTGACCCTTACACACCTGGATTCCCTTCCTTCAATCACACTCAGTTTCCACCATCTCGGTCATCAGGATTGCCTAATATACCTGTCCAGACAATCTCCAGAGCTGCTGCAGAAAAGCTGTTTGGGAATATGGAAGGAGACTGTCCCTCTGACTGGAAAACAGACTCTACATGTAGGATGGTAACCTCAGAAAGCAAGAATGTGAAGCTCACTGTGAGCAATGTGCTGAAAGAGATAAAAATTCTTAACATCTTTGGAGTTATTAAAGGCTTTGTAGAACCAGATCACTATGTTGTAGTTGGGGCCCAGAGAGATGCATGGGGCCCTGGAGCTGCAAAATCCGGTGTAGGCACAGCTCTCCTATTGAAACTTGCCCAGATGTTCTCAGATATGGTCTTAAAAGATGGGTTTCAGCCCAGCAGAAGCATTATCTTTGCCAGTTGGAGTGCTGGAGACTTTGGATCGGTTGGTGCCACTGAATGGCTAGAGGGATACCTTTCGTCCCTGCATTTAAAGGCTTTCACTTATATTAATCTGGATAAAGCGGTTCTTGGTACCAGCAACTTCAAGGTTTCTGCCAGCCCACTGTTGTATACGCTTATTGAGAAAACAATGCAAAATGTGAAGCATCCGGTTACTGGGCAATTTCTATATCAGGACAGCAACTGGGCCAGCAAAGTTGAGAAACTCACTTTAGACAATGCTGCTTTCCCTTTCCTTGCATATTCTGGAATCCCAGCAGTTTCTTTCTGTTTTTGCGAGGACACAGATTATCCTTATTTGGGTACCACCATGGACACCTATAAGGAACTGATTGAGAGGATTCCTGAGTTGAACAAAGTGGCACGAGCAGCTGCAGAGGTCGCTGGTCAGTTCGTGATTAAACTAACCCATGATGTTGAATTGAACCTGGACTATGAGAGGTACAACAGCCAACTGCTTTCATTTGTGAGGGATCTGAACCAATACAGAGCAGACATAAAGGAAATGGGCCTGAGTTTACAGTGGCTGTATTCTGCTCGTGGAGACTTCTTCCGTGCTACTTCCAGACTAACAACAGATTTCGGGAATGCTGAGAAAACAGACAGATTTGTCATGAAGAAACTCAATGATCGTGTCATGAGAGTGGAGTATCACTTCCTCTCTCCCTACGTATCTCCAAAAGAGTCTCCTTTCCGACATGTCTTCTGGGGCTCCGGCTCTCACACGCTGCCAGCTTTACTGGAGAACTTGAAACTGCGTAAACAAAATAACGGTGCTTTTAATGAAACGCTGTTCAGAAACCAGTTGGCTCTAGCTACTTGGACTATTCAGGGAGCTGCAAATGCCCTCTCTGGTGACGTTTGGGACATTGACAATGAGTTTTAAggcgcgcc |

***Supplementary Table 2. Primer sequence***

|  | Forward primer (5′→3′) | Reverse primer (5′→3′) |
| --- | --- | --- |
| IGF2BP2  TFRC  STEAP3  DMT1  Cycline A1  Cycline A2  Cycline B1  Cycline D1  Cycline D2  Cycline E1  METTL4  Actin  M^6^A-TFRC  GAPDH | AGTGGAATTGCATGGGAAAATCA  GTTGAATTGAACCTGGAC  GCCGCAGAGCCACCAA  TGTGTTCTACTTGGGTTGGCA  AAGATAACGACGGGAAGAGCG  CTGGTGGTCTGTGGTCTGTGA  AACTTTCGCCTGAGCCTATTTT  GCTGCGAAGTGGAAACCATC  CTGTCTCTGACCGCAAGCAT  AAGGAGCGGGACACCATGA  TATCCCTCTTGGTCTGTGGAG  CTCCATCCTGGCCTCGCTGT  GTGAGAGACTGGCAGGAACC  AATCCCATCACCATCTTCCAG | CAACGGCGGTTTCTGTGTC  AAGTAGCACGGAAGAAGT  CTCCCACCACCACTTTGAAA  CTGGCTCTGATGGCTACCTG  TCCCCAGCCCCCAATAAAAG  TAAACTTCTTGGATGCCAGTCT  TTGGTCTGACTGCTTGCTCTT  CCTCCTTCTGCACACATTTGAA  GGTGGGTACATGGCAAACTTAAA  ACGGTCACGTTTGCCTTCC  ACCTTCGTAGGGCTTTTTGTG  GCTGTCACCTTCACCGTTCC  TTGAGATCCAGCCTCACGAG  AAATGAGCCCCAGCCTTC |

***Supplementary Table 3. Primary and secondary antibodies and dilution ratio***

| Target  IGF2BP2  TFRC  STEAP3  DMT1  Cycline A1  Cycline A2  Cycline B1  Cycline D1  Cycline D2  Cycline E1  METTL4  β-actin  anti-mouse HRP  secondary antibody  anti-rabbit HRP  secondary antibody | Company  Proteintech,China  Immunoway,USA  Proteintech,China  Proteintech,China  Immunoway,USA  Immunoway,USA  Immunoway,USA  Immunoway,USA  Immunoway,USA  Immunoway,USA  Bioss,China  Proteintech,China  Immunoway,USA  Immunoway,USA | Cat.No.  11601-1-AP  YT5374  17186-1-AP  20507-1-AP  YT1168  YN1560  YT1169  YT1172  YT1174  YT1176  bs-18851R  60008-1-Ig  RS0001  RS0002 | Dilution ratio  1:3000  1:1000  1:2000  1:1000  1:1000  1:1000  1:1000  1:1000  1:1000  1:1000  1:1000  1:5000  1:5000  1:500 |
| --- | --- | --- | --- |

***Supplementary Figure***

Figure S1. Statistics of GO enrichment analysis, from TCGA(https://www.aclbi.com/static/index.html#/tcga).


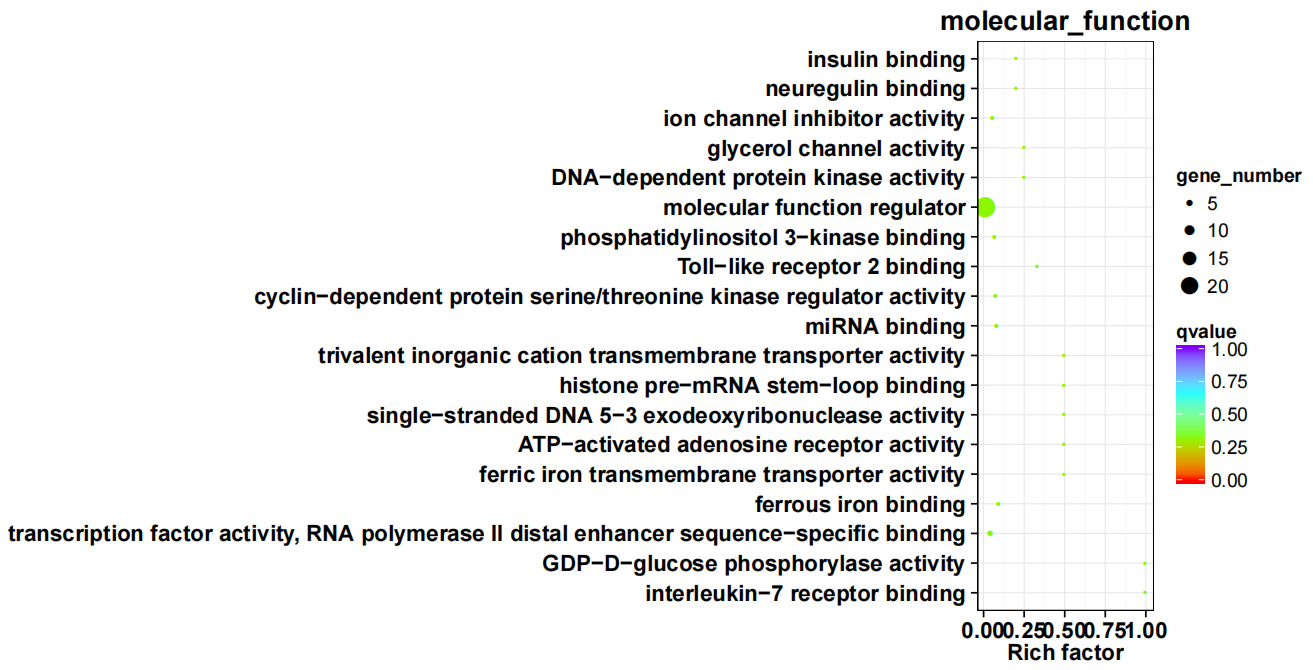

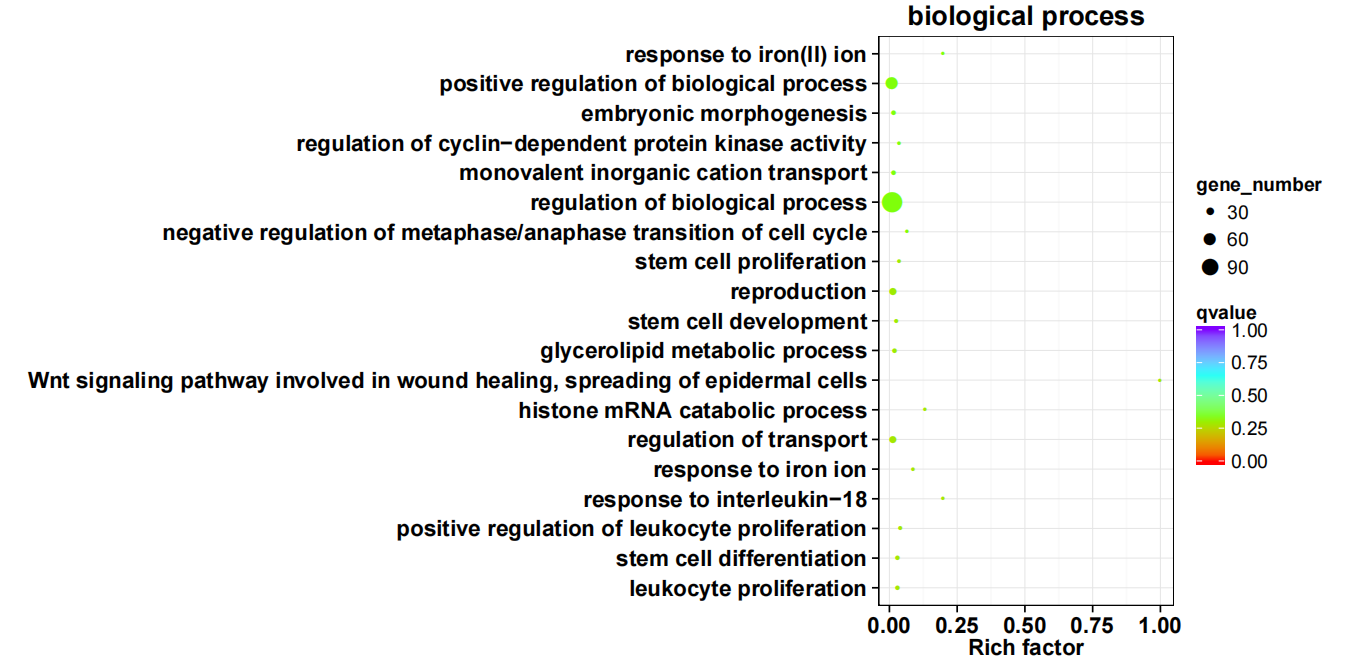

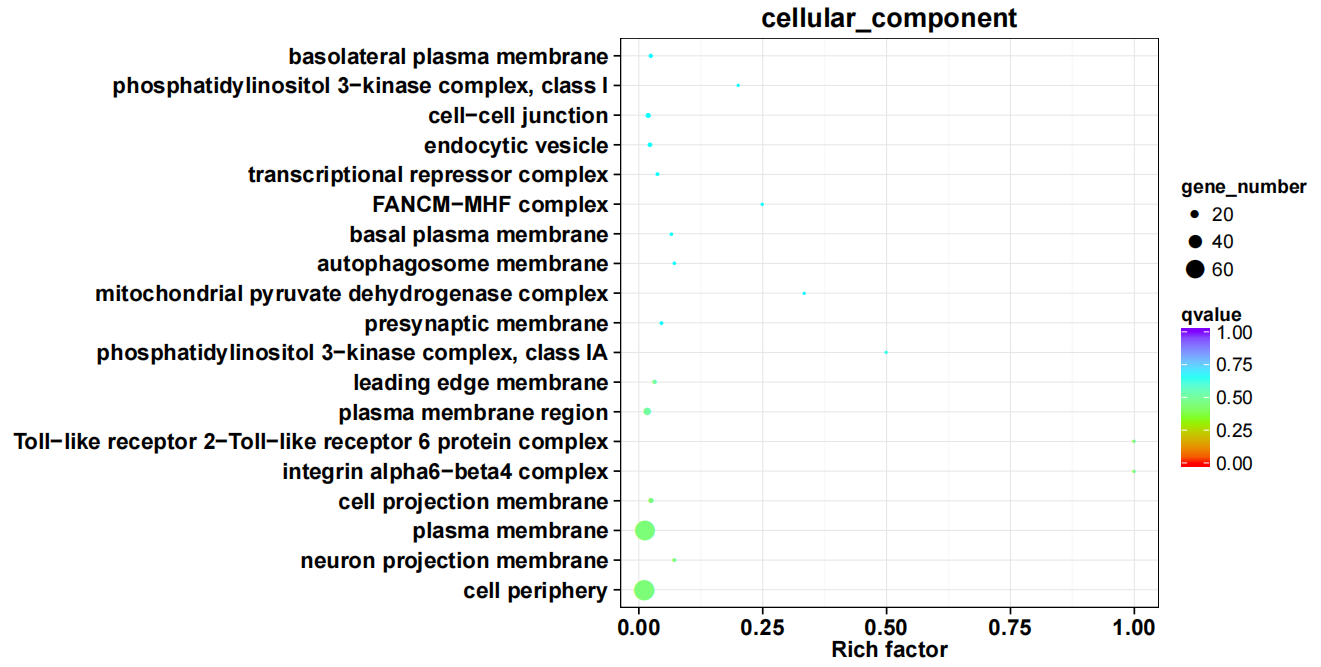


Figure S2 .The binding of IGF2BP2 to TFRC was verified by RIP assay. Each value represents the mean ± SD for triplicate samples (Student t test). **** *P* < 0.0001.
